# Influence of Ceramic Membrane Surface Characteristics on the Flux Behavior of a Complex Fermentation Broth

**DOI:** 10.3390/membranes11060402

**Published:** 2021-05-28

**Authors:** Nicolas A. P. Maguire, Mehrdad Ebrahimi, Rong Fan, Sabine Gießelmann, Frank Ehlen, Steffen Schütz, Peter Czermak

**Affiliations:** 1 Institute of Bioprocess Engineering and Pharmaceutical Technology, University of Applied Sciences Mittelhessen, 35390 Giessen, Germany; nicolas.andrew.peter.maguire@lse.thm.de (N.A.P.M.); mehrdad.ebrahimi@lse.thm.de (M.E.); 2 Fraunhofer Institute for Molecular Biology and Applied Ecology (IME), Project Group Bioresources, 35392 Giessen, Germany; 3 Faculty of Biology and Chemistry, Justus-Liebig University of Giessen, 35390 Giessen, Germany; 4 MANN+HUMMEL GmbH, 71636 Ludwigsburg, Germany; Sabine.Giesselmann@mann-hummel.com (S.G.); Frank.Ehlen@mann-hummel.com (F.E.); Steffen.Schuetz@mann-hummel.com (S.S.)

**Keywords:** ceramic membrane, biomass separation, agro-industrial residue, *Kluyveromyces lactis*, surface roughness, composition separating layer, scanning electron microscopy

## Abstract

The valorization of agro-industrial residues using yeasts as biocatalysts requires efficient methods for biomass separation. Filtration with ceramic membranes is suitable for this task, however, the challenge of flux decline and the unavoidable cleaning must be taken into account. We investigated the filtration of fermentation broth and its components using tubular microfiltration and ultrafiltration membranes, and hollow-fiber ultrafiltration membranes, with cut-offs of 30 and 200 nm. The steady-state flux was limited by fouling under comparable wall shear stress conditions but increased when the wall shear stress was higher. Single-component filtration with two 30 nm tubular ultrafiltration membranes, whose average surface roughness ranged from 1.0 to 3.9 µm, showed that smoother surfaces experience less biomass fouling under more intense hydrodynamic conditions. Furthermore, we showed experimentally and by scanning electron microscopy in filtration with 30 nm tubular membranes that the thickness of the first separation layer is responsible for the degree of irreversible resistance caused by the deposition of organic material in the membrane pores. The thickness of this layer should therefore be minimized without compromising mechanical stability.

## 1. Introduction

The biotechnological processing of agro-industrial residues enables the valorization of waste streams. There is global interest in the use of such residues as a raw material for the production of fuels, biological preservatives, pharmaceuticals, and other high-value goods, but there is still a need for more research [1]. Bacteria and yeasts can be used as biocatalysts for a wide range of applications involving the conversion of residues into high-value products. The yeast *Kluyveromyces lactis* is particularly suitable for this task because it can utilize diverse nutrient sources, produce large quantities of recombinant proteins, and has a generally regarded as safe (GRAS) status [2].

Following fermentation, the liquid and solid components of the broth must be separated. Centrifugation, depth filtration or membrane filtration are suitable for this separation task, but membrane filtration is the least expensive in terms of upfront costs and the most scalable. Additionally, sterile membrane filtration is mandatory when using genetically modified organisms to ensure the removal of cells [3]. However, one disadvantage of membranes is the high cost of consumables, so it is important to reuse the membranes as much as possible [4]. With a life span of more than 10 years and the ability to tolerate harsh chemicals and extreme pH, ceramic membranes are ideal for a long service life [5,6]. The reusability of such membranes requires us to address the challenges of flux decline due to fouling and unavoidable cleaning, thus restoring membrane permeability.

A comprehensive review of cleaning methods for ceramic membranes was recently published [7]. The susceptibility of membranes to fouling (and thus the loss of flux) is influenced by the feed composition, hydrodynamic conditions, and membrane properties. The latter include surface roughness, the thickness of the separation layer, charge, hydrophobicity, and functional groups on the surface [8]. The surface roughness is particularly relevant when the size of the filtered particles or colloids is similar to or smaller than the asperities on the surface [9,10]. When particles or colloids are smaller than the asperities, the probability of deposition in the valleys increases [11]. However, the filtration resistance of a membrane increases as the separating layers become thicker [12,13].

Fouling analysis is required for the characterization of filtration processes. This includes detecting the onset of fouling and the analysis of factors related to fouling, helping to explain fouling mechanisms and the quantification or prediction of fouling behavior [14]. For example, the biofouling potential of industrial fermentation broth during microfiltration (MF) was investigated by scanning electron microscopy (SEM) and scanning acoustic microscopy (SAM), which allow the qualitative determination of fouling mechanisms, together with the resistance-in-series (RIS) and combined pore-blockage and cake filtration models, which facilitate quantitative analysis [15]. The fouling of MF [16], ultrafiltration (UF) [17], and reverse osmosis (RO) [18] membranes, among others, has been analyzed qualitatively by SEM, whereas RIS models are used regularly for the quantitative analysis of fouling involving biological foulants such as enzymes [19], protein aggregates [20], viruses [21], and bacteria [22].

The factors that affect membrane fouling, including surface roughness and separation layer thickness, are often investigated using model solutions such as bovine serum albumin (BSA) [23]. For the separation of yeast biomass, most studies use baker’s yeast (*Saccharomyces cerevisiae*), typically freeze-dried and rehydrated even though the results tend to differ compared to freshly cultivated cells [16,24]. Here we considered the industrial strain *Kluyveromyces lactis* and studied the influence of membrane surface properties on fouling during the separation of biomass from the fermentation broth. The cells were cultivated in a medium derived from agro-industrial residues containing corn steep powder and whey and were filtered using ceramic membranes with different pore sizes (30 nm and 200 nm) and geometries (tubular and hollow fiber). Here, we focused on analyzing the surface roughness and the composition of the separation layer for two 30 nm cut-off tubular membranes and determined their individual susceptibility to fouling by proteins and yeast cells. We also investigated the influence of operational parameters such as crossflow velocity (CFV) on filtration efficiency and fouling.

## 2. Materials and Methods

### 2.1. Strain and Fermentation Medium

For biomass production, we used *K. lactis* strain GG799 (New England Biolabs, Frankfurt, Germany). The fermentation medium consisted of 3.0% (*w*/*v*) corn steep powder SOLULYS 095E (Roquette Frères, Lestrem, France), 0.6% (*w*/*v*) heated and crystallized sweet whey powder (Bayrische Milchindustrie, Landshut, Germany) and 150 mM citrate-phosphate buffer (pH 5), comprising 10.2 g L^−1^ citric acid monohydrate (AppliChem, Darmstadt, Germany) and 14.6 g L^−1^ disodium hydrogen phosphate (Merck, Darmstadt, Germany). To reduce the natural particle load, the suspensions of corn steep and whey powder were passed through a 100 nm Al_2_O_3_ filter before use. 

### 2.2. Cultivation of K. lactis

We used a 5-L bioreactor system (Applikon Biotechnology, Delft, The Netherlands) with a working volume of 4 L for the cultivation of *K. lactis*. The bioreactor was operated at 30 °C, with an air aeration rate of 3 L min^−1^ and an agitation speed of 800 rpm. The pH was not controlled. To avoid foaming, we added 0.01% (*v*/*v*) Struktol J673A (Schill & Seilacher, Hamburg, Germany) before inoculation. The culture was inoculated with a glycerol cryo-stock to an optical density of ΔOD_600_ = 0.1. 

### 2.3. Harvest of Medium and Biomass

The culture was harvested after 25 h, having reached the stationary phase. The fermentation broth was drawn aseptically and cooled immediately in an ice bath. The biomass and the medium were separated by centrifugation (17,207× *g* for 10 min at 4 °C) in a Sigma 6–16 KS centrifuge equipped with an 11,650 rotor and six 13,650 cups (Sigma Laborzentrifugen, Osterode am Harz, Germany). The medium was passed through a 0.22-µm polyethersulfone filter (Corning, New York, NY, USA) and stored at 4 °C. To remove residual medium, the biomass pellet was resuspended in 154 mM NaCl and centrifuged as described above. The supernatant was discarded, and the pellet was resuspended in 150 mM citrate-phosphate buffer (pH 5) and stored at 4 °C.

### 2.4. Filtration Setup

**Figure 1:**
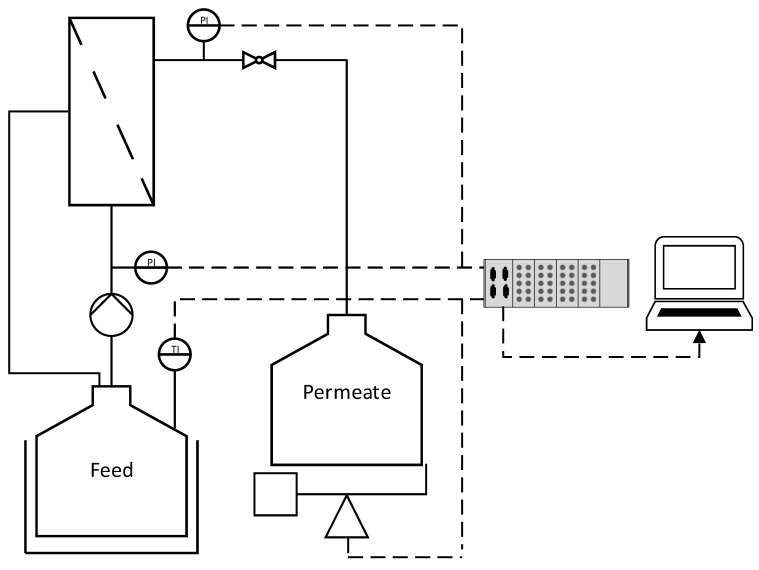
The filtration setup used in this study. Dashed lines indicate data transmission. P = pressure sensor. T = temperature sensor.

The technical data for the ceramic membranes used in this study are summarized in Table 1. All membranes were manufactured and kindly provided by MANN+HUMMEL (Ludwigsburg, Germany), except for the monochannel 200 nm MF membrane, which was purchased from Atech Innovations (Gladbeck, Germany). The feed solution (1.5 L) was circulated using an FCPA 80B-4/HE rotary vane pump (AFT, Rosstal, Germany). The temperature of the feed vessel was measured using a PT-100 sensor and was maintained at 25 ± 1 °C by immersion in a water bath. The transmembrane pressure (TMP) was adjusted using a manual ball valve located behind the membrane module and was measured using two type-401001 sensors (JUMO Mess- und Regeltechnik, Vienna, Austria) in front and behind the membrane module. The feed volume flow was measured using an SM6000 magnetic-inductive flow sensor (ifm electronic, Essen, Germany). The permeate flow was measured gravimetrically using a DS 8K.05 balance (Kern & Sohn, Balingen, Germany). Filtrations were carried out in total recycle mode (TRM), and the permeate was regularly recycled to the feed. Data were recorded using a LABmanager 1 and the corresponding software LabVision v2.9 (both from HiTec Zang, Herzogenrath, Germany). The filtration setup is shown in Figure 1.

### 2.5. Estimation of Intrinsic Membrane Resistance and Irreversible Resistance

The intrinsic membrane resistance R_m_ [m^−1^] was determined before filtration and after chemical cleaning. It was calculated from the pure water flux at a CFV of 0.8 m s^−1^ (1.6 m s^−1^ for the 9C HF membrane) and TMPs of 0.1–1.1 bar at 25 °C using Equation (1):

(1) $$R_{m}\;=\;\frac{\mathrm{TMP}}{\eta \;\cdot \;J_{w}}$$

where R_m_ is the intrinsic membrane resistance [m^−1^], *η* is the permeate dynamic viscosity [Pa s], TMP is the transmembrane pressure during filtration [Pa], and J_w_ is the pure water permeate flux [m^3^ m^−2^ s^−1^]. The viscosity of water is shown in Table 2. To calculate the irreversible fouling resistance R_n, irrev_ (Equation (2)), the fouled membrane was flushed with deionized water at 50 °C for 20 min using the same process parameters described above and with the permeate valve closed, then with fresh deionized water at 50 °C for 20 min under the same process parameters with the permeate valve open. The index n indicates the fouling resistance of the fermentation broth, medium, or yeast cells, as appropriate.

(2) $$R_{n,\;\mathrm{irrev}}\;=\;\frac{\mathrm{TMP}}{\eta \;\cdot \;J_{w}}\;-\;R_{m}$$

The pure water flux of the rinsed membrane was then measured again as described above. The irreversible resistance corresponds to the resistance that can only be removed by chemical cleaning and not water rinsing. 

### 2.6. Investigation of Total, Fouling, and Reversible Resistance

We determined the total resistance R_ferm, tot_ of each membrane by filtrations with fermentation broth. The feed consisted of 0.75 L medium and biomass to a concentration of 2.5 g L^−1^ cell dry weight (CDW) mixed with 150 mM citrate-phosphate buffer (pH 5) in a total volume of 1.5 L. When the feed was fully mixed, the pH was adjusted to pH 5 with 37% hydrochloric acid (Carl Roth, Karlsruhe, Germany). Before filtration, the feed was circulated in the system for 10 min at 0.5 bar TMP, a CFV of 0.8 m s^−1^ (1.6 m s^−1^ for the 9C_HF membrane) and a temperature of 25 °C with the permeate valve closed. The valve was then opened, and filtration was carried out with manual back-recycling of permeate, until a steady-state flux was established. The total resistance R_ferm, tot_ and the fouling resistance R_ferm_ were calculated using Equation (3). Single-component resistances were investigated using the 30 nm tubular UF membranes 7C_s and 7C_r. Feed preparation and filtration were then carried out using medium or biomass as described above, and the corresponding resistances R_yeast_ and R_medium_ were calculated using Equation (3).

(3) $$R_{n}\;=\;R_{n,\;\mathrm{tot}}-R_{m}\;=\;\frac{\mathrm{TMP}}{\eta \cdot J_{\mathrm{ss}}}-R_{m}$$

where J_ss_ is the respective steady-state flux, and the index n indicates the fouling resistance of the fermentation broth, medium, or yeast cells, as appropriate. The respective viscosities are shown in Table 2.

The reversible resistance was calculated by subtracting the irreversible resistance from the fouling resistance using Equation (4).

(4) $$R_{n,\;\mathrm{rev}}\;=\;R_{n}\;-\;R_{n,\;\mathrm{irrev}}$$

### 2.7. Chemical Cleaning

To restore the initial filtration performance of each membrane after estimating the irreversible resistance, chemical cleaning was carried out with 1% (*w*/*v*) P3 Ultrasil 14 (Ecolab Deutschland, Monheim am Rhein, Germany) at 60 °C, 0.5 bar TMP, and a CFV of 0.8 m s^−1^ (1.6 m s^−1^ for the 9C HF membrane) for 2 h with the permeate valve open. The system was then rinsed with deionized water until the permeate returned to neutral. 

### 2.8. Reynolds Number and Wall Shear Stress Calculations

To characterize the flow regime, we calculated the Reynolds number (Re) using Equation (5):

(5) Re = ρ⋅CFV⋅dη

where *ρ* is the density [kg m^−3^], CFV is the crossflow velocity [m s^−1^], d is the inner channel diameter [m], and *η* is the dynamic feed viscosity [Pa s]. The wall shear stress *τ*_w_ [Pa] was calculated using Equation (6):

(6) $$\tau_{w}\;=\lambda \cdot \rho \cdot \frac{{\mathrm{CFV}}^{2}}{2}$$

where *λ* is the drag coefficient calculated as a function of the Reynolds number using the Blasius correlation (Equation (7)). This is applied in the presence of turbulent flow and hydraulic smooth pipes (2320 < Re < 10^5^).

(7) λ =0.316⋅Re−0.25

When the roughness of the surface is higher, the drag coefficient is calculated for the transition area using the Colebrook equation. However, the roughness encountered in this study was below the transition area [25].

### 2.9. Viscosity Measurement

Viscosity was determined using a Haake RS 300 rheometer (Thermo Fisher Scientific, Waltham, MA, USA) fitted with a plate-cone measuring device (2° angle) equipped with a Haake DC30 thermostat (Thermo Fisher Scientific) for temperature control.

### 2.10. Particle Size Measurements

The particle size distribution of *K. lactis* cells after harvest was measured in the range 0.02–2000 µm with a Mastersizer 2000 (Malvern Panalytical, Malvern, UK) using the laser diffraction method. For each sample, ten measurements were taken with automatic parameter calculation.

### 2.11. Measurement of Biomass Concentration

Biomass concentrations were determined by measuring the optical density ΔOD_600_ and CDW. Absolute OD_600_ values were measured with a BioSpectrometer basic (Eppendorf, Hamburg, Germany). The samples were diluted with 150 mM citrate-phosphate buffer (pH 5) to OD_600_ < 0.5. The ΔOD_600_ was calculated from the difference between sample absorbance and blank, multiplied by the dilution factor. The CDW was determined by weighing the washed pellet from 2 mL samples after drying for 24 h at 80 °C.

### 2.12. Bradford Assay

Total protein was measured using Bradford reagent prepared from 0.1 g L^−1^ Coomassie Brilliant Blue (Applichem), 47.5 mL L^−1^ ethanol (Applichem), and 136 mL L^−1^ 75% orthophosphoric acid (VWR, Radnor, PA, USA). BSA (Carl Roth) was used as a standard. For the colorimetric assay, 30 µL of sample, blank or standard, was mixed with 270 µL of Bradford reagent and incubated for 5 min. The absorbance was measured at 450 and 590 nm using a Synergy HT plate reader (BioTek, Winooski, VT, USA). The absorbance ratio 590/450 nm was used for quantification [26]. 

### 2.13. Surface Characterization of Membranes 7C_s and 7C_r

Membrane surface roughness was estimated by the manufacturer (MANN+HUMMEL) according to standard operating procedures using a VK-X110 3D laser scanning confocal microscope and the associated software v2.8.0.0 (both from Keyence, Neu-Isenburg, Germany). The 50× magnification data were processed by locally estimated scatterplot smoothing (LOESS) using OriginPro 2019b (Origin Lab, Northampton, MA, USA). The resulting data were used to calculate the arithmetic mean roughness parameter R_a_ using Equation (8):

(8) $$R_{a}=\;\frac{1}{N}\overset{N}{\underset{j=0}{\sum }}\left|Z_{j}\right|$$

where Z_j_ is the current value at the measurement point, and N is the number of measurement points. The thickness of the separation layers was determined by the manufacturer (MANN+HUMMEL) according to standard operating procedures again using a Keyence VK-X110 3D laser scanning confocal microscope. Briefly, the membrane was broken into fragments and images of the interface were captured at 50× magnification. The thickness of each layer was calculated using Keyence software v2.8.0.0.

### 2.14. SEM Images for Fouling Analysis

Fouling of the separating layers after filtration was characterized using an EVO LS 10 scanning electron microscope (Carl Zeiss, Oberkochen, Germany) in high-vacuum mode. After filtration, the membranes 7C_s and 7C_r (45 mm in length) were rinsed with water and then broken into fragments and dried for 24 h at 50 °C. Before analysis, the surface was sputtered with gold to prevent surface charge build-ups.

## 3. Results

### 3.1. Characterization of the Filtration Feed 

**Figure 2:**
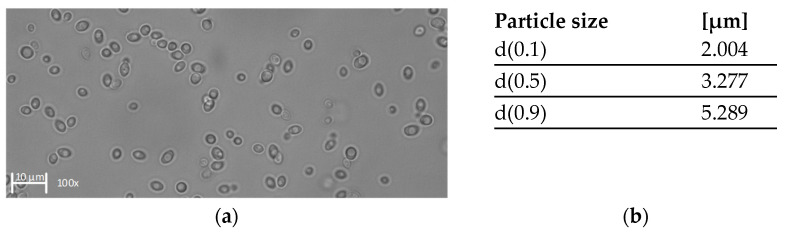
Characterization of *K. lactis* cells after harvest. (**a**) Image of *K. lactis* in 150 mM citrate-phosphate buffer (pH 5) captured by light microscopy at 100× magnification. (**b**) Calculated d-values based on the volume related particle size measurement.

We characterized the feed in terms of total protein concentration, viscosity, and the size distribution of *K. lactis* cells. Figure 2 shows the size distribution along with an image of the *K. lactis* cells after harvest, captured by light microscopy. The viscosity of the filtration feed is shown in Table 2.

The total protein concentration was 1.1 g L^−1^ ± 0.12 g L^−1^. The size distribution of *K. lactis* was unimodal with a median value of 3.277 µm. We found that 10% of the cells were at least 2.004 µm in diameter, and 90% of the cells were below 5.289 µm in diameter. The pronounced unimodal size distribution was also supported by light microscopy, revealing oval cells predominantly of a similar size.

### 3.2. Comparison of Pure Water Fluxes

**Figure 3:**
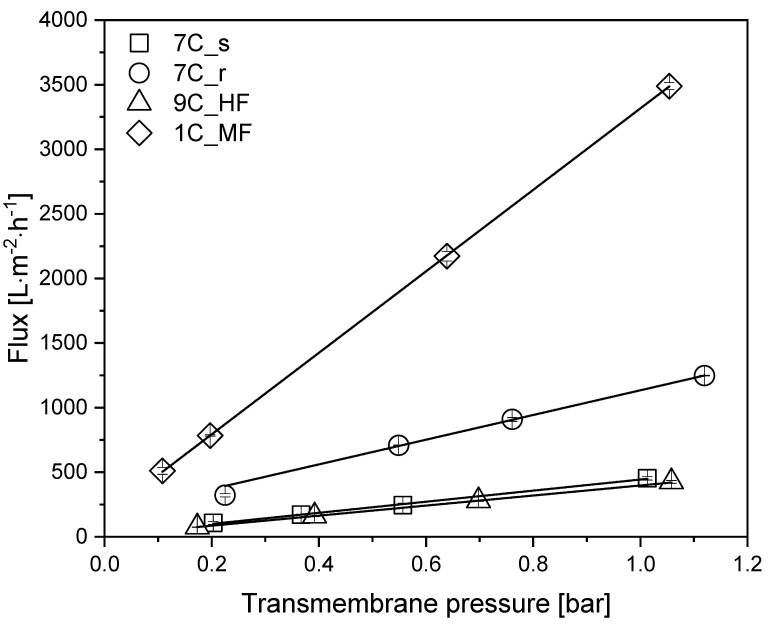
TMP-dependent pure water flux of each membrane. Solid lines indicate linear fits. Data are means ± standard deviations (n = 3). The coefficient of determination R^2^ for each fit was >0.98.

To determine the intrinsic membrane resistance and the restoration of the initial flux after cleaning, we measured the TMP-dependent water flux of each membrane (Table 1) in the pressure range 0.1–1.1 bar at 25 °C (Figure 3).

All membranes showed a linear relationship between flux and TMP within the indicated pressure range. At 0.5 bar, the highest pure water flux of 1751 L m^−2^ h^−1^ was reached by the MF membrane 1C_MF. Among the 30 nm UF membranes, the highest pure water flux of 628 L m^−2^ h^−1^ was achieved by tubular membrane 7C_r, followed by tubular membrane 7C_s (227 L m^−2^ h^−1^) and the hollow-fiber membrane 9C_HF (203 L m^−2^ h^−1^). Membrane 7C_r therefore showed an approximately 3-fold higher flux than the other membranes with the same pore size. After each filtration, the membranes were cleaned as described in Section 2.7, and the initial flux was always restored (data not shown).

### 3.3. Characterization of Surface Roughness and the Separating Layers of Tubular UF Membranes

**Figure 4:**
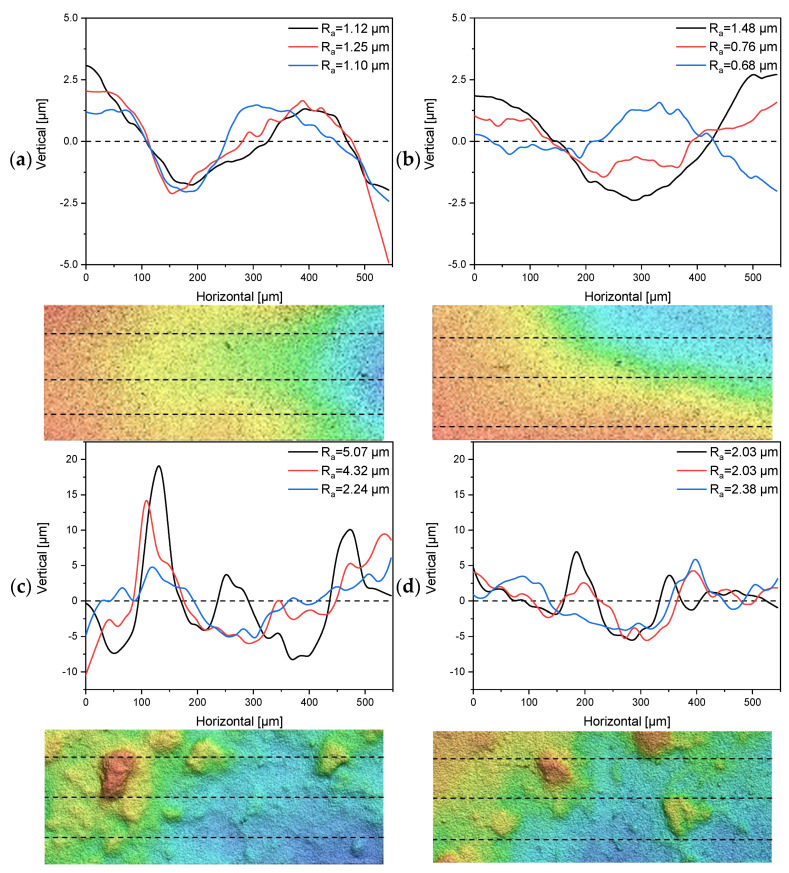
One-dimensional profiles for the determination of surface roughness and topographical images (200 µm × 550 µm) from multiple line measurements of the tubular UF membranes 7C_s and 7C_r. Dashed lines indicate the profile location. (**a**,**b**) Profiles and topographical images from two randomly-selected segments of membrane 7C_s. (**c**,**d**) Profiles and topographical images from two randomly-selected segments of membrane 7C_r.

The 30 nm tubular ceramic membranes 7C_s and 7C_r were characterized in terms of their surface roughness and the number and thickness of separating layers. The 1D profiles of surface roughness measurements for each membrane and a topographic image indicating the measured lines are shown in Figure 4. The composition of the separating layers is shown in Table 3. See Supplementary Material S1.1 for the corresponding microscopy images.

The 1D profiles of surface roughness showed a clear distinction in terms of R_a_ values between the 7C_s and 7C_r membranes. The mean R_a_ value calculated from the three-line measurements of membrane 7C_s was 1.15 µm ± 0.08 µm for the first segment (Figure 4a) and 0.97 µm ± 0.4 µm for the second segment (Figure 4b). The total mean R_a_ value was therefore 1.07 µm ± 0.3 µm. The topographic images of membrane 7C_s show a smooth decline in height from left to right, whereas the images of membrane 7C_r show distinct hills and valleys distributed across the investigated area. The mean R_a_ value calculated from the three-line measurements of membrane 7C_r was 3.88 µm ± 1.47 µm for the first segment (Figure 4c) and 2.15 µm ± 0.2 µm for the second segment (Figure 4d). The total mean R_a_ value was therefore 3.01 µm ± 1.33 µm. Even a small shift in the position of the lines would influence the R_a_ value for membrane 7C_r, but our data clearly show that membrane 7C_s has a smoother and more even surface than 7C_r.

The number of separating layers is three for membrane 7C_s but only two for membrane 7C_r. Furthermore, the first (cutoff-determining) separating layer is approximately 3-fold thicker for 7C_s compared to 7C_r (Table 3). The pore size of the remaining layers was not investigated. Notably, the support material for membrane 7C_r is a mixture of Al_2_O_3_ and TiO_2_, whereas that for membrane 7C_s is entirely Al_2_O_3_.

### 3.4. Performance of the Membranes with Fermentation Broth

**Figure 5:**
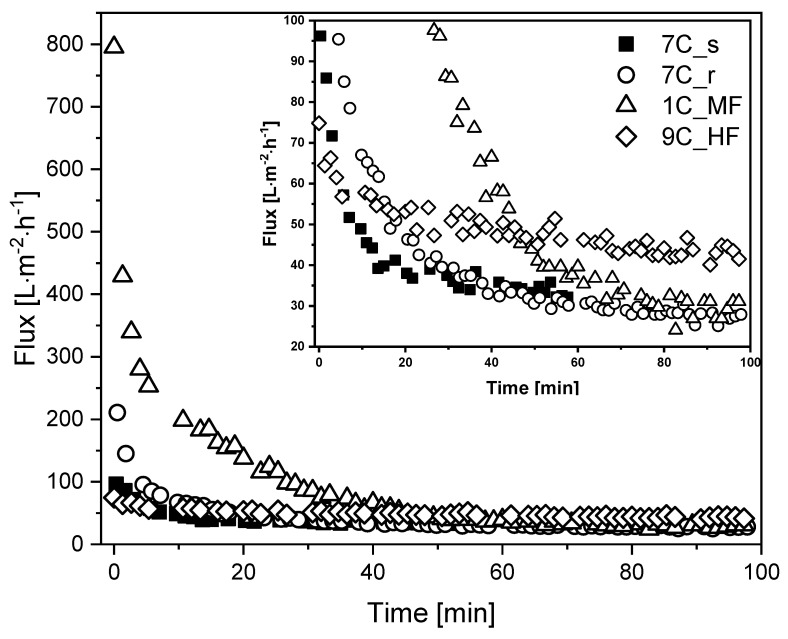
Flux curves during the filtration of fermentation broth with different membranes (TMP = 0.5 bar, temperature = 25 °C). The CFV was 0.8 m s^−1^ for all membranes except 9C_HF (CFV = 1.6 m s^−1^). An enlargement of the graph covering the low-flux region is shown in the upper right corner for clarity.

We investigated the membrane flux in total recycle mode (TRM) using fermentation broth as the feed. Figure 5 shows that steady-state flux J_ss_ was achieved after 60–100 min, with the highest J_ss_ observed for the UF hollow-fiber membrane. 

The MF membrane 1C_MF showed the highest J_0_ (795 L m^−2^ h^−1^), followed by 7C_r (211 L m^−2^ h^−1^), 7C_s (96 L m^−2^ h^−1^), and 9C_HF (75 L m^−2^ h^−1^). Flux reduction was least severe for the hollow-fiber membrane (~40% loss, falling to 43 L m^−2^ h^−1^) whereas the other membranes lost more than 50% flux within the first 20 min. The tubular membranes reached a comparable J_ss_ of 27–33 L m^−2^ h^−1^ regardless of the pore size. Given the differences in channel diameter and CFV, the filtrations involving the hollow-fiber membrane (channel diameter = 2 mm, CFV = 1.6 m s^−1^) and tubular membranes (channel diameter = 6 mm, CFV = 0.8 m s^−1^) differ in terms of flow regime and wall shear stress. Accordingly, Re = 2731 and *τ*_w_ = 56.0 Pa for the hollow-fiber membrane in contrast to Re = 4096 and *τ*_w_ = 12.6 Pa for the tubular membranes. The retention of total protein and biomass was also monitored for the first 60 min of each filtration. We observed no differences in total protein concentration between the feed at the beginning of filtration, the retentate and permeate. The biomass concentration in the retentate varied between 90% and 100% during filtration, and no biomass was detected in the permeate samples. The deposition of biomass on the membrane or in the dead spaces of the filtration apparatus was therefore negligible. The Bradford assay results and biomass monitoring data are provided in Supplementary Material S1.2.

### 3.5. Calculated Resistances for the Filtration of Fermentation Broth

**Figure 6:**
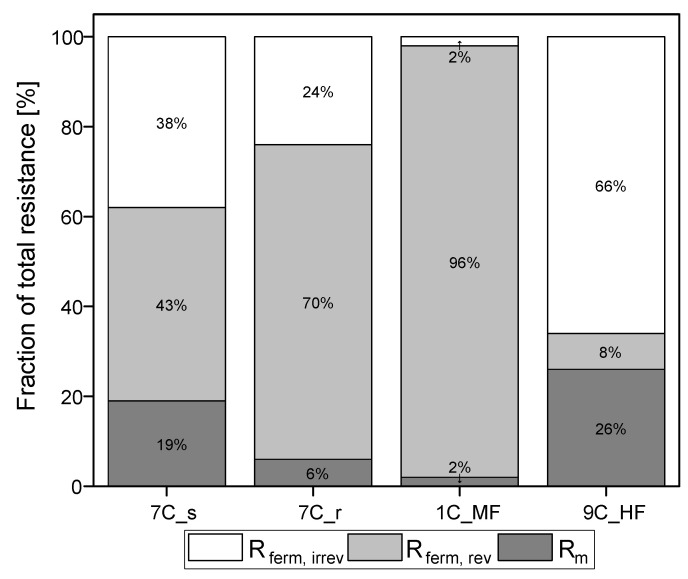
Partition of resistances caused by the filtration of fermentation broth. The sum of the reversible and irreversible resistance is R_ferm_. The sum of R_m_ and R_ferm_ is R_ferm, tot_. The 100% values correspond to a total resistance R_ferm, tot_ of 5.04 × 10^12^ m^−1^ for 7C_s, 5.52 × 10^12^ m^−1^ for 7C_r, 5.38 × 10^12^ m^−1^ for 1C_M, and 3.81 × 10^12^ m^−1^ for 9C_HF.

Figure 6 shows the membrane resistances for the filtration runs discussed above and partitions them into reversible and irreversible resistances. The intrinsic membrane resistance R_m_ was calculated from the values shown in Figure 3 (TMP = 0.5 bar). Given the differences in R_m_ despite similar overall resistance R_ferm, tot_, there are nevertheless also clear differences in R_ferm_ when comparing the tubular membranes. The absolute values of resistances caused by the filtration of fermentation broth are provided in Supplementary Material S1.3.

Irreversible resistance as a proportion of total resistance ranged from 66% for the ceramic UF hollow-fiber membrane to 2% for the MF membrane 1C_MF, with the 7C_s and 7C_r membranes showing intermediate values of 38% and 24%, respectively. The membrane with the highest steady-state flux therefore also showed the highest proportion of irreversible resistance. In contrast, membrane 1C_MF (with the largest pore size of 200 nm) showed the lowest proportion of irreversible resistance. Among the 30 nm membranes (7C_s, 7C_r and 9C_HF), the partitioning of resistance varied independently of pore size. 

### 3.6. Filtration of Medium-Free K. lactis Cell Suspension

**Figure 7:**
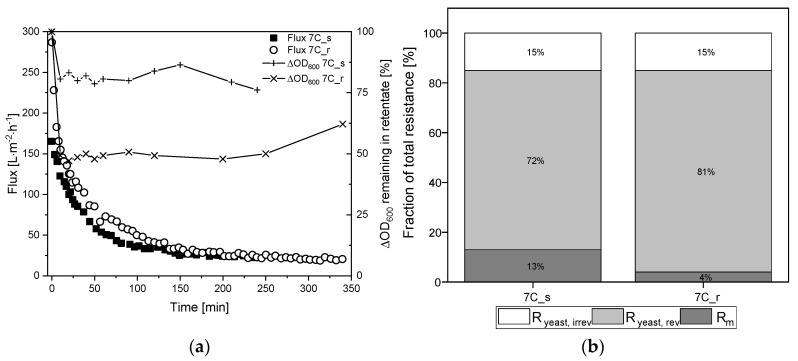
Filtration flux curves and calculated percentage resistances of *K. lactis* cells (TMP = 0.5 bar, CFV = 0.8 m s^−1^, temperature = 25 °C) using 30 nm tubular UF membranes 7C_s and 7C_r. (**a**) The flux curve (J_ss_ after 240–350 min) and the remaining biomass in the retentate. (**b**) Partition of resistances caused by the filtration of yeast cells. The sum of the reversible and irreversible resistance is R_yeast_. The sum of R_m_ and R_yeast_ is R_yeast, tot_. The 100% values correspond to a total resistance R_yeast, tot_ of 7.08 × 10^12^ m^−1^ for 7C_s, and 8.79 × 10^12^ m^−1^ for 7C_r.

To investigate the influence of surface roughness and the number and thickness of separating layers in more detail, the fermentation broth was fractionated into the two main components medium and yeast cells. Figure 7a shows the TRM flux curves using *K. lactis* cells as a feed at a CFV of 0.8 m s^−1^ and the biomass remaining in the retentate based on the ΔOD_600_. The corresponding percentage resistances are shown in Figure 7b. The absolute values of resistances caused by the filtration of *K. lactis* cells are provided in Supplementary Material S1.3.

The filtration of 2.5 g L^−1^ *K. lactis* cells reached steady-state flux after 240 min for membrane 7C_s (J_ss_ = 24 L m^−2^ h^−1^) and 350 min for membrane 7C_r (J_ss_ = 20 L m^−2^ h^−1^), corresponding to 85% and 93% flux loss, respectively. These fluxes were lower than those observed for the complete fermentation broth, and it took considerably longer to reach J_ss_. The irreversible fraction of total resistance was 15% for both membranes, although the biomass concentration in the retentate differed between the membranes we used. The biomass in the retentate was reduced by ~25% for membrane 7C_s but ~50% for membrane 7C_r. At the end of filtration, immediate flushing of the membrane for 5 min at a CFV of 0.8 m s^−1^ with the permeate valve closed restored the initial amount of biomass for both membranes.

### 3.7. Fouling Control by Regulating the CFV

**Figure 8:**
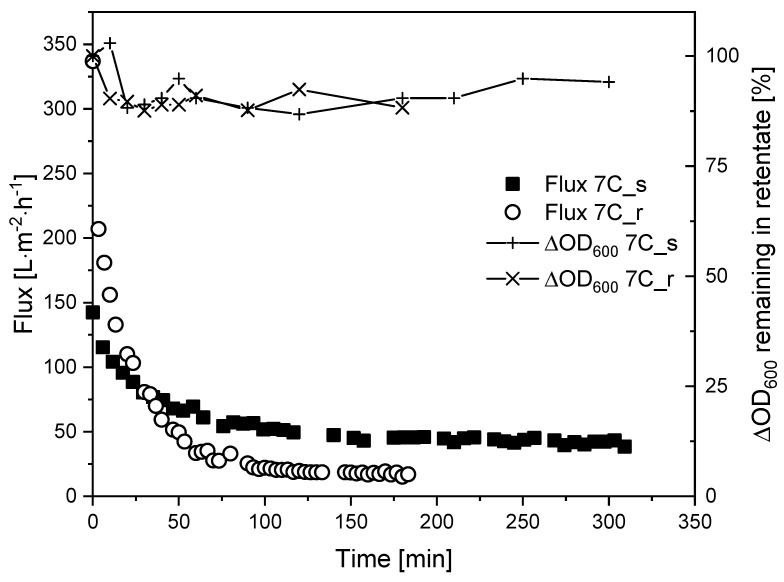
Flux curves and biomass remaining in the retentate during the filtration of *K. lactis* cells (TMP = 0.5 bar, CFV = 1.1 m s^−1^, temperature = 25 °C) using membranes 7C_s and 7C_r.

Next, we investigated whether fouling can be controlled by regulating the CFV. We therefore repeated the TRM filtrations with *K. lactis* cell suspensions at a higher CFV of 1.1 m s^−1^, and the corresponding flux curves are shown in Figure 8.

Increasing the CFV from 0.8 m s^−1^ (*τ*_w_ = 12.6 Pa) to 1.1 m s^−1^ (*τ*_w_ = 21.8 Pa) did not appear to affect the J_ss_ of membrane 7C_r (17 L m^−2^ h^−1^) but increased the J_ss_ of membrane 7C_s to 40 L m^−2^ h^−1^. In both filtrations at CFV = 1.1 m s^−1^, the reduction of biomass in the retentate was 90–100%, comparable to the filtrations with fermentation broth. 

### 3.8. Filtration of Cell-Free Medium

**Figure 9:**
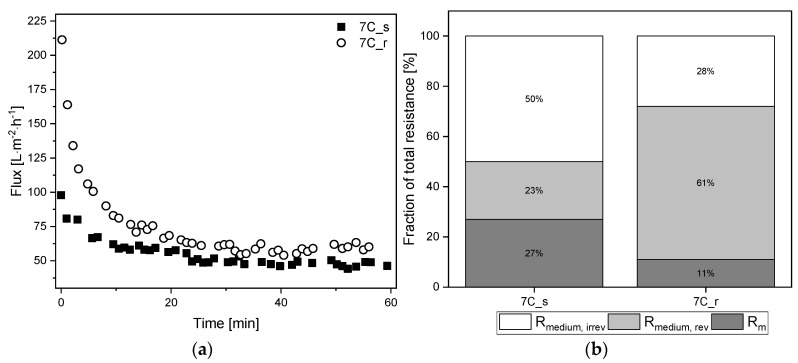
Filtration flux curves and calculated percentage resistances of cell-free medium (TMP = 0.5 bar, CFV = 0.8 m s^−1^, temperature = 25 °C) using 30 nm tubular UF membranes 7C_s and 7C_r. (**a**) The flux curve (J_ss_ begins at 60 min). (**b**) Partition of resistances caused by the filtration of cell-free medium. The sum of the reversible and irreversible resistance is R_medium_. The sum of R_m_ and R_medium_ is R_medium, tot_. The 100% values correspond to a total resistance R_medium, tot_ of 3.44 × 10^12^ m^−1^ for 7C_s, and 3.08 × 10^12^ m^−1^ for 7C_r.

The influence of surface roughness and the number and thickness of the separation layers was also tested on the cell-free medium in a TRM filtration with the same process parameters used for the complete fermentation broth (TMP = 0.5 bar, CFV = 0.8 m s^−1^). Figure 9a shows the TRM flux curves using the cell-free medium as a feed solution, with a steady-state flux achieved within 60 min for both membranes. The corresponding percentage resistances are shown in Figure 9b. The absolute values of resistances caused by the filtration of cell-free medium are provided in Supplementary Material S1.3.

After 60 min, the tubular UF membranes reached comparable steady-state fluxes of J_ss_ = 57 L m^−2^ h^−1^ for 7C_r (flux loss of 27%) and J_ss_ = 48 L m^−2^ h^−1^ for 7C_s (flux loss of 50%). Although the total resistance of 7C_s was higher, the resulting fouling resistance R_medium_ was lower due to the higher intrinsic membrane resistance. There were also clear differences between the reversible and irreversible resistance fractions, with the smoother membrane 7C_s showing a higher proportion of irreversible fouling (50%) than the rougher membrane 7C_r (28%). The irreversible resistance was therefore lower for membrane 7C_r during the filtration of cell-free medium and fermentation broth.

### 3.9. Fouling Analysis by SEM

**Figure 10:**
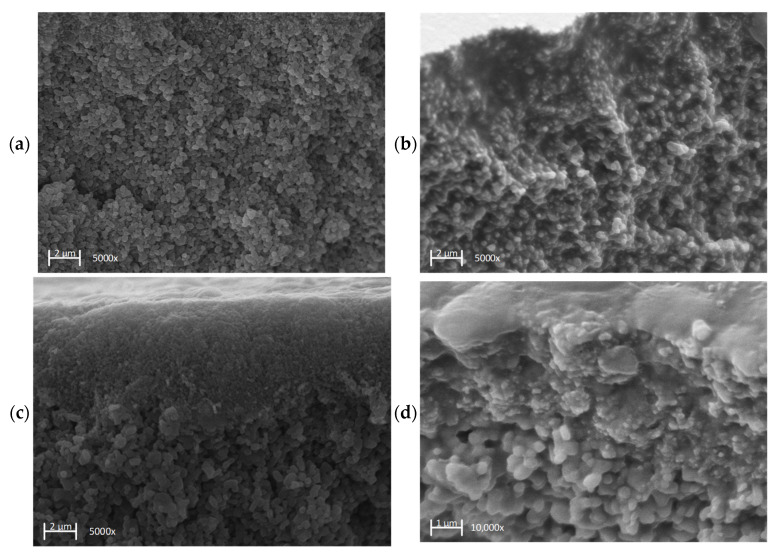
SEM images of the fractured edge of the tubular UF membranes 7C_s and 7C_r in clean and fouled conditions, the latter after the filtration of fermentation broth. (**a**) First separation layer of the clean 7C_s membrane. (**b**) First separation layer of the fouled 7C_s membrane. (**c**) First and second separation layers of the clean 7C_r membrane. (**d**) First and second separation layers of the fouled 7C_r membrane.

To investigate the fouling effects within the separating layers in more detail, clean and fouled samples of membranes 7C_s and 7C_r were examined by SEM. Images of the fracture edge of both membranes in both conditions are shown in Figure 10.

The images of the clean membranes show distinct particulate structures for the first separating layer (Figure 10a,c) and in the case of 7C_r also the second separating layer, with larger particles (Figure 10c). The latter also reveals the differing thickness of the separation layers (Table 3). The images of the fouled membranes (Figure 10b,d) show organic material between the inorganic membrane Al_2_O_3_ particles. This organic material densely fills the inter-particle gaps in the first separation layer and is also present in the second separation layer but is less densely packed (Figure 10d).

## 4. Discussion

MF membranes with pore sizes of 0.1–0.2 µm are often used to separate cells from culture medium. However, particles that are close to the pore size are likely to access the pores, leading to a severe fouling. *S. cerevisiae* (cell size ≤ 10 µm) is often used as a model for membrane filtration, but *K. lactis* is smaller [27], and our data confirmed a size distribution of approximately 2–5 µm. We therefore focused on the investigation of fouling on the surface of 30 nm membranes during the filtration of medium containing *K. lactis* cells. The water permeability of membranes is determined by the pore size, overall porosity, and the thickness of the separation layers [28]. In our experiments, the membrane with the largest pore size of 200 nm achieved a higher pure water flux than the 30 nm membranes, but there were clear differences among the UF membranes. A closer look at tubular UF membranes 7C_s and 7C_r revealed differences in the thickness and number of separation layers. The thickness of the layers has a direct impact on water permeability and is the decisive factor for the observed differences [28,29,30]. Reducing the number and thickness of layers can increase the surface roughness [31]. This interaction is due to the manufacturing process of ceramic membranes, which are often produced in a dip-coating process in which intermediate layers and a final separating layer are coated to the support material [32,33]. The support material provides the mechanical stability, while the intermediate layers reduce the pore size and creates a relatively homogeneous surface. The final separation layer consists of the smallest pores and determines the separation characteristics of the membrane [32]. The relationship between the number of layers, which increases the cost, and the surface roughness must be taken into account in the manufacturing process. The actual number of layers also depends on the production itself, the technique used is confidential information of the respective manufacturer. Furthermore, differences in water permeability caused by surface roughness have been studied for UF and nanofiltration (NF) membranes. For ceramic UF membranes, roughness was shown not to influence the pure water permeability [11], whereas studies of polysulfone NF membranes showed that roughness had a positive effect on the pure water permeability. Rougher surfaces feature valleys with lower membrane resistance, and most of the mass flow through the membranes passes through these valleys [34]. In our case, the membrane with the rougher surface also featured the thinner outer separating layer. Two different effects would therefore occur in the valleys: thinning of the outer separation layer, which decreases membrane resistance, and general thinning of the membrane, further decreasing membrane resistance.

The wall shear stress was used to compare the performance of different membrane pore sizes and geometries. This parameter has already been used successfully to compare ceramic hollow-fiber and tubular membranes with different inner channel diameters [35]. During the filtration of fermentation broth under comparable process conditions, all three tubular membranes (30 nm and 200 nm) reached a comparable J_ss_ of 27–33 L m^−2^ h^−1^ regardless of the pore size, suggesting that steady-state flux was strictly limited by fouling effects occurring under the prevailing hydrodynamic conditions. Similar results were observed using BSA as a feed solution (CFV = 9.5 cm s^−1^) with identically constructed flat-sheet polymeric reverse osmosis (RO), NF, and UF membranes [36]. Despite differences in J_0_ reflecting the diverse membrane properties, all membranes reached an almost identical J_ss_ probably because flux is primarily influenced by interactions between the fouled membrane and the foulant [36]. 

Increasing the wall shear stress reduces the formation of a fouling layer on the membrane surface and thus increases flux [37,38]. In filtrations with spent sulphite liquor using 8 nm hollow fiber membranes, the effect of increasing flux by increasing wall shear stress was investigated. The authors showed an increase in flux by a factor of ~5 with increasing wall shear stress from 6–130 Pa and consequentially a reduction in fouling [35]. In our filtrations, the wall shear stress was ~4.5-fold higher on the hollow-fiber membrane than the tubular membranes due to the smaller inner channel diameter and higher CFV, suggesting that the higher J_ss_ was positively influenced by enhanced particle back-transport. Taking the reversible and irreversible resistance into consideration, the hollow-fiber UF membrane (with the highest J_ss_ ) also showed the highest irreversible resistance (66%). However, the MF membrane 1C_MF with the largest pore size of 200 nm showed a considerably lower irreversible resistance of 2%. A high proportion of reversible fouling, as we observed for membrane 1C_MF, indicates the formation of external deposits of material that can be removed easily by rinsing or filtration breaks [14]. Higher wall shear stress reduces the deposition of external material on the hollow-fiber membrane, thus leading to other fouling effects such as internal pore clogging and thus a higher proportion of irreversible resistance. The higher shear stress therefore minimized reversible fouling without influencing the irreversible fouling [39]. The MF membrane experienced substantially less irreversible resistance compared to the other membranes because the yeast cells reduce membrane fouling caused by proteins. A combination of BSA and *S. cerevisiae* limits the fouling of internal membrane structures compared to BSA alone because the yeast cells form a layer that acts like a pre-filter [40,41]. Our protein concentration data revealed little to no protein retention. Further experiments are required to study simultaneous biomass separation and the retention or transmission of specific proteins.

The filtration of medium-free cell suspensions using membranes 7C_s and 7C_r at a CFV of 0.8 m s^−1^ resulted in a comparable J_ss_ , indicating that surface roughness does not affect the J_ss_ in the range studied. J_0_ was higher for cell suspensions than fermentation broth, but the J_ss_ was ~10 L m^−2^ h^−1^ lower, and the time to reach J_ss_ was longer. This indicates that other fouling mechanisms are responsible, ruling out the direct comparison and summation of resistances, as per the RIS model. Figure 7b shows an irreversible resistance of 15% for membranes 7C_s and 7C_r after the filtration of medium-free cell suspensions, showing that differences in irreversible resistance arising from the filtration of fermentation broth must depend on components in the supernatant. Furthermore, although we observed differences in biomass in the retentate during filtration (Figure 7a), these differences did not affect the flux. This probably reflects a tradeoff between the thickness of the top layer of cells and the thickness of the separation layer. The smaller number of cells in the retentate of membrane 7C_r should ensure that more cell deposition results in greater resistance compared to 7C_s. However, this is offset by the thinner separating layer of membrane 7C_r, thus resulting in a similar J_ss_. Given that almost all the biomass could be recovered after filtration by rinsing the membrane, the biomass deposited on the membranes was not a dense cake layer. Indeed, additional filtrations at a CFV of 1.1 m s^−1^ revealed clear differences in steady state fluxes. The J_ss_ increased from 24 L m^−2^ h^−1^ at 0.8 ms^−1^ to 40 L m^−2^ h^−1^ at 1.1 m s^−1^ for the smooth surface of membrane 7C_s, but the CFV had a negligible effect on the rougher surface of 7C_r (J_ss_ = 20 L m^−2^ h^−1^ at 0.8 m s^−1^ and 17 L m^−2^ h^−1^ at 1.1 m s^−1^). Increasing the CFV reduces superficial membrane fouling and thus increases flux during the filtration of biological suspensions [42]. In addition, smoother membrane surfaces can promote flux by reducing susceptibility to fouling [11,43,44]. The effects we observed at a CFV of 1.1 m s^−1^ indicate that only the smooth membrane has this effect, although the biomass remaining in the retentate increased to 90–100% in both cases. This provides evidence that less foreign material is deposited on the membrane surface, thus limiting the resistance caused by cells. The CFV-dependent behavior of the two membranes should therefore be investigated in more detail.

Comparing the filtration data for the fermentation broth (cells plus medium) and the cell-free medium revealed further interesting phenomena. The J_0_ of the tubular UF membrane 7C_s and that of 7C_r were comparable, regardless of the feed solution, but a higher J_ss_ was achieved when the resistance caused by yeast cells was absent. We propose that the initial drop in flux during filtrations with cell-free medium is accompanied by the formation of a gel layer, hence the additional presence of cells in the fermentation broth only slightly increases the resistance. A gel layer is not formed during the filtration of cell suspensions without medium, but the cells form a loose filter cake that builds up until the J_ss_ is reached, thus causing more resistance than the combination of a gel layer and cells. Based on these observed resistances, a theoretical resistance can be attributed to the yeast cells even though this cannot be determined experimentally. For the 30 nm membranes, we calculated yeast cell resistances of 1.53 × 10^12^ m^−1^ for 7C_s and 2.44 × 10^12^ m^−1^ for 7C_r. Although the mass fluxes differ only slightly, the resistances indicate that the smooth membrane 7C_s tends to be less exposed to fouling by the cells, which is likely to reflect the surface roughness.

The 1D profiles and topographic images of 7C_r revealed an irregular surface featuring peaks and valleys. Single line measurements revealed R_a_ values of ≥5 µm, which is in the same size range as *K. lactis* cells and thus increases the probability of particle deposition in the valleys of the asperities of the membrane surface. The deposition of particles in valleys leading to more fouling has already been described as a possible cause for the reduction of permeate flows [45,46]. The fraction of irreversible resistance increased slightly during the filtration of cell-free medium compared to fermentation broth, probably because (as discussed above) the yeast cells may act as a pre-filter. The greater irreversible resistance of the 7C_s membrane, which has a smoother surface as well as more numerous and thicker separation layers compared to the 7C_r membrane, was also evident in the filtration of cell-free medium. Medium components that penetrate the membrane pores must therefore be responsible. The difference in the irreversible fraction could be due to surface roughness or the composition of the separating layers, and this can be investigated by SEM. We found that organic material was present at the highest density in the first layer. For the smoother membrane with the thicker first layer, a large amount of densely packed organic material accumulated in the pores, and such material was also present in the second layer, albeit at a lower density. Based on our results, we therefore assume that the thickness of the first layer (and thus the larger surface area in this layer) is responsible for the formation of the higher irreversible fraction due to interfacial interactions between product components and the membrane. Similarly, for the filtration of skimmed milk using membranes that differed only in the thickness of the outer separation layer, the membranes with the thinner separation layer were characterized by lower levels of irreversible resistance caused by the feed [30].

The membrane properties responsible for fouling can be determined more precisely by investigating additional properties such as the zeta potential of the membrane and feed, membrane chemistry, pore size distribution, porosity, and contact angle in addition to the surface roughness and composition of the separation layers discussed herein [47].

## 5. Conclusions

We characterized two 30 nm tubular ceramic UF membranes in terms of surface roughness and the structure of the separating layers and investigated the influence of these properties on the separation of *K. lactis* biomass from a medium composed of agro-industrial residues. We also demonstrated the use of different ceramic membranes varying in pore size and geometry for the same application. Little to no protein was retained by the membranes. The surface roughness of the membranes influenced the degree of biomass fouling with increasing wall shear stress, and the membrane with a smoother surface showed the best performance. The nature of the separating layers affected the formation of irreversible resistances. Our data indicate that the number and thickness of the separating layers should be minimized while ensuring membrane stability, particularly in the case of filtrations with a long duration. Resistances that can only be removed by chemical cleaning have a substantial negative influence on long-term filtration performance and thus on overall productivity and economic efficiency.

## Figures and Tables

**Table 1:** Properties of the ceramic membrane used in this study.

Property	Membrane			
	7C_s	7C_r	1C MF	9C HF
Geometry	tubular	tubular	tubular	hollow fiber
Support material	Al_2_O_3_	Al_2_O_3_/TiO_2_	Al_2_O_3_	Al_2_O_3_
Separating layers	Al_2_O_3_	Al_2_O_3_	Al_2_O_3_	Al_2_O_3_
Cut-off [nm]	30	30	200	30
Length [mm]	225	230	225	200
Number of channels	7	7	1	9
Inner diameter of each channel [mm]	6	6	6	2
Filtration area [m^2^]	2.97 × 10^−2^	3.03 × 10^−2^	0.42 × 10^−2^	1.13 × 10^−2^
Cross-flow area [m^2^]	1.98 × 10^−4^	1.98 × 10^−4^	2.83 × 10^−5^	2.83 × 10^−5^

**Table 2:** Viscosity of each solution used in filtration experiments at 25 °C.

Solution	Viscosity [mPa∙s]
Medium + yeast cells	1.17
Yeast cells (2.5 g/L)	1.11
Medium	1.12
Pure water	0.89

**Table 3:** Number and thickness of Al_2_O_3_ separating layers in the 7C_s and 7C_r membranes.

Property	Membrane	
	7C_s	7C_r
Number of layers	3	2
Thickness of first layer	9.57 ± 1.37 µm	2.51 ± 0.49 µm
Thickness of second layer	26.41 ± 1.99 µm	43.42 ± 6.19 µm
Thickness of third layer	49.09 ± 2.80 µm	-

## Supplementary Materials

The following are available online at https://www.mdpi.com/article/10.3390/membranes11060402/s1. Figure S1: Images of two randomly selected segments of membrane 7C_s at 50× magnification. The layers are named in parentheses, Figure S2: Images of two randomly selected segments of membrane 7C_s at 50× magnification. The layers are named in parentheses, Figure S3: Bradford assay showing the amount of protein in the retentate and permeate and the remaining optical density in the retentate of the four membranes during the filtration of fermentation broth: (a) 7C_s, (b) 7C_r, (c) 1C_MF, and (d) 9C_HF; Table S1: Calculated resistances caused by the filtration of the fermentation broth. The values in parentheses correspond to the fraction of the total resistance. The sum of Rm and Rferm corresponds to Rferm, tot. The sum of the reversible and irreversible resistance results in Rferm. Table S2: Calculated resistances caused by the filtration of *K. lactis* cells. The values in parentheses correspond to the proportion of the total resistance. The sum of R_m_ and R_yeast_ corresponds to R_yeast, tot_. The sum of the reversible and irreversible resistance results in R_yeast_; Table S3: Calculated resistances caused by the filtration of cell-free medium. The values in parentheses correspond to the proportion of the total resistance. The sum of R_m_ and R_medium_ corresponds to R_medium, tot_. The sum of the reversible and irreversible resistance results in R_medium_.
