# Supplementary material for: Influence of Ceramic Membrane Surface Characteristics on the Flux Behavior of a Complex Fermentation Broth
Source: Membranes (Basel). 2021 May 28;11(6):402. doi: 10.3390/membranes11060402 (PMC8229547; doi:10.3390/membranes11060402)
Supplement: Supplementary file 1 [file membranes-11-00402-s001.zip › membranes-1220449-supplementary.pdf]

membranes

MDPI

Supplementary Material

Influence of Ceramic Membrane Surface Characteristics on the Flux Behavior of a Complex Fermentation Broth

Nicolas A. P. Maguire [1,2,3], Mehrdad Ebrahimi [1], Rong Fan [1,2,*], Sabine Gießelmann [4], Frank Ehlen [4], Steffen Schütz [4] and Peter Czermak [1,2,3,*]

1 Institute of Bioprocess Engineering and Pharmaceutical Technology, University of Applied Sciences Mittelhessen, 35390 Giessen, Germany; nicolas.andrew.peter.maguire@lse.thm.de (N.A.P.M.); mehrdad.ebrahimi@lse.thm.de (M.E.)
2 Fraunhofer Institute for Molecular Biology and Applied Ecology (IME), Project Group Bioresources, 35392 Giessen, Germany
3 Faculty of Biology and Chemistry, Justus-Liebig University of Giessen, 35390 Giessen, Germany
4 MANN+HUMMEL GmbH, 71636 Ludwigsburg, Germany; Sabine.Giesselmann@mann-hummel.com (S.G.); Frank.Ehlen@mann-hummel.com (F.E.); Steffen.Schuetz@mann-hummel.com (S.S.)
* Correspondence: rong.fan@ime.fraunhofer.de (R.F.); peter.czermak@lse.thm.de (P.C.); Tel.: +49-641-972-19312 (R.F.); +49-641-309-2650/2551 (P.C.)

S1. Supplementary Material

S1.1. Images of membranes 7C_s and 7C_r captured by light microscopy.

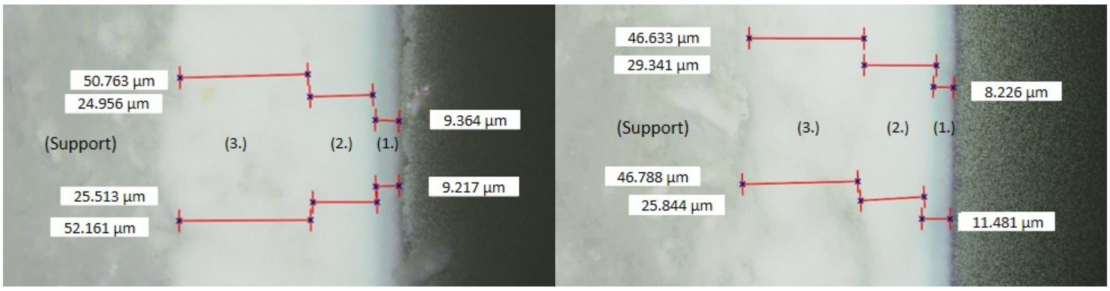

Figure S1. Images of two randomly selected segments of membrane 7C_s at 50× magnification. The layers are named in parentheses.

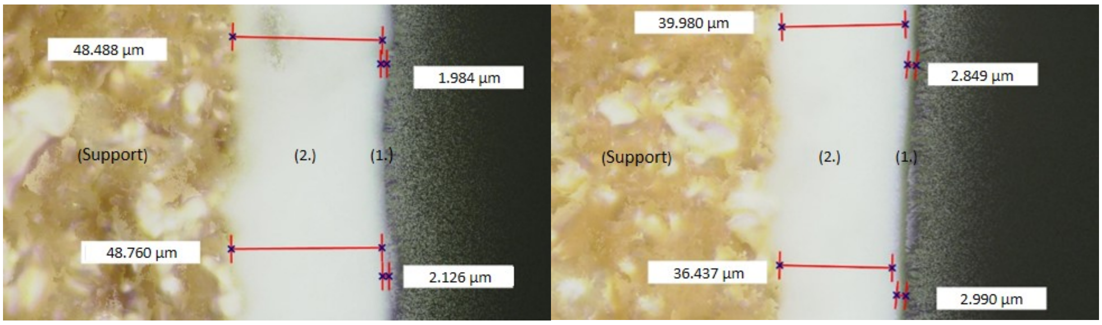

Figure S2. Images of two randomly selected segments of membrane 7C_s at 50× magnification. The layers are named in parentheses.

S1.2. Bradford assay and optical density data

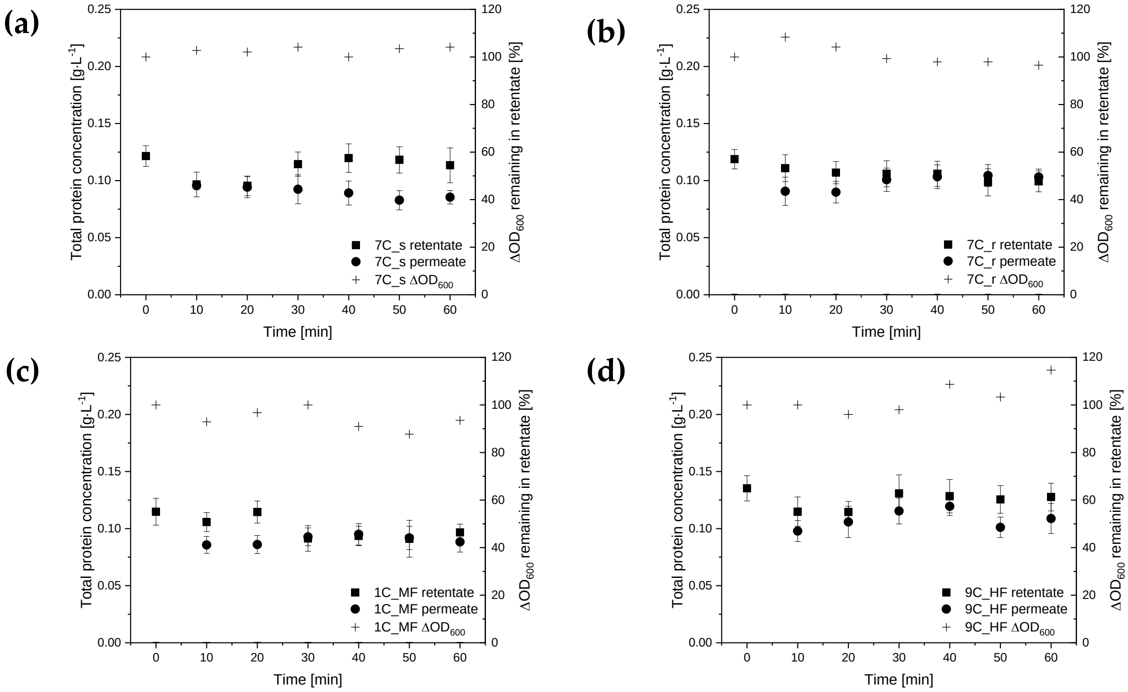

Figure S3. Bradford assay showing the amount of protein in the retentate and permeate and the remaining optical density in the retentate of the four membranes during the filtration of fermentation broth: (a) 7C_s, (b) 7C_r, (c) 1C_MF, and (d) 9C_HF.

S1.3. Calculated resistances caused by filtrations of fermentation broth, *K. lactis cells* and cell-free medium.

Table S1. Calculated resistances caused by the filtration of the fermentation broth. The values in parentheses correspond to the fraction of the total resistance. The sum of R_m and R_{ferm} corresponds to $R_{ferm, tot}$. The sum of the reversible and irreversible resistance results in R_{ferm}.

	Resistance [1 × 10^{12} m^{-1}]			
	**7C_s**	**7C_r**	**1C_MF**	**9C_HF**
$\mathbf{R_{ferm, tot}}$	5.04 (100%)	5.52 (100%)	5.38 (100%)	3.81 (100%)
$\mathbf{R_m}$	0.922 (19%)	0.350 (6%)	0.116 (2%)	0.993 (26%)
$\mathbf{R_{ferm}}$	4.05 (81%)	5.17 (94%)	5.26 (98%)	2.82 (74%)
$\mathbf{R_{ferm, irrev}}$	1.90 (38%)	1.33 (24%)	0.120 (2%)	2.52 (66%)
$\mathbf{R_{ferm, rev}}$	2.14 (43%)	3.83 (70%)	5.14 (96%)	0.292 (8%)

$R_{ferm, tot}$ = total resistance; R_m = intrinsic membrane resistance; R_{ferm} = resistance due to fermentation broth; $R_{ferm, irrev}$ = irreversible resistance fraction of R_{ferm}; $R_{ferm, rev}$ = reversible resistance fraction of R_{ferm}.

Table S2. Calculated resistances caused by the filtration of *K. lactis* cells. The values in parentheses correspond to the proportion of the total resistance. The sum of R_m and R_{yeast} corresponds to $R_{yeast, tot}$. The sum of the reversible and irreversible resistance results in R_{yeast}.

	Resistance [1 × 10^{12} m^{-1}]	
	**7C_s**	**7C_r**
$\mathbf{R_{yeast, tot}}$	7.08 (100%)	8.79 (100%)
$\mathbf{R_m}$	0.922 (13%)	0.350 (4%)
$\mathbf{R_{yeast}}$	6.15 (87%)	8.44 (96%)
$\mathbf{R_{yeast, irrev}}$	1.06 (15%)	1.31 (15%)
$\mathbf{R_{yeast, rev}}$	5.10 (72%)	7.14 (81%)

$R_{yeast, tot}$ = total resistance; R_m = intrinsic membrane resistance; R_{yeast} = resistance due to yeast components; $R_{yeast, irrev}$ = irreversible resistance fraction of R_{yeast}; $R_{yeast, rev}$ = reversible resistance fraction of R_{yeast}.

Table S3. Calculated resistances caused by the filtration of cell-free medium. The values in parentheses correspond to the proportion of the total resistance. The sum of R_m and R_{medium} corresponds to $R_{medium, tot}$. The sum of the reversible and irreversible resistance results in R_{medium}.

	Resistance [1 × 10^{12} m^{-1}]	
	**7C_s**	**7C_r**
$\mathbf{R_{medium, tot}}$	3.44 (100%)	3.08 (100%)
$\mathbf{R_m}$	0.922 (27%)	0.350 (11%)
$\mathbf{R_{medium}}$	2.52 (73%)	2.73 (89%)
$\mathbf{R_{medium, irrev}}$	1.73 (50%)	0.86 (28%)
$\mathbf{R_{medium, rev}}$	0.79 (23%)	1.87 (61%)

$R_{medium, tot}$ = total resistance; R_m = intrinsic membrane resistance; R_{medium} = resistance due to medium components; $R_{medium, irrev}$ = irreversible resistance fraction of R_{medium}; $R_{medium, rev}$ = reversible resistance fraction of R_{medium}.
